# The Code of Ethics

**Published:** 1894-01

**Authors:** M. DeCausey

**Affiliations:** Sealy


For Texas Medical Journal.

### "THE CODE OF ETHICS."

BY M. DE CAUSEY, M. D., SEALY.

Read at the meeting of the Austin County Medical Association, at Bellville, Sept. 19, 1893.

THE HYPPOCRATIC OATH was a masterpiece of ethics in its day and time, and was admirably suited to its atmosphere of primitive honesty and sterling honor. But like the *favored* of past ages, it exists now only in fossil remains, as it is no longer adapted to the surroundings of the nineteenth century, and its degenerate successor, "The Code of Ethics," is amenable to the inexorable law of the survival of the fittest, and the conditions for its existence, in its present state, are no longer favorable, and it, too, must soon succumb to the buffetings of time, unless it is fostered with greater care than it now receives.

It is a truth which none of you will question, that laymen of all classes have more intelligent ideas of almost anything else than they have of medicine, and many of them, regarding the physician, as at best, but a necessary evil, are somewhat invidious and disposed to distrust his noblest motives and aspirations, as in some way having a sinister bearing on themselves.

The traitorous apostates from "The Code of Ethics" have assiduously instilled into the receptive minds of these people the idea that "The Code of Ethics," so far from being their only charter of safety against the machinations of charlatanism, is the quintessence of charlatanry itself. A professional rhodomontade having for its real object the attainment, by the "regulars," of the exclusive privilege of extorting unreasonable and unmerited fees from their unlucky and helpless patrons.

The physician, standing on the principles of ethics, is too honest, too modest, to meet the expectations of an exacting, unreasonable and over-credulous public. The oracular, the mysterious, the miraculous, the impossible, are now, as they have ever been, the enchanters of the human mind, and charlatans have not been backward in taking advantage of this fact in the furtherance of their own sordid purposes, by blatant professions of their own wonderful achievement, and by disparaging and slandering the knowledge and methods of regular physicians in a manner that would outrage common sense, if common sense were exercised; but the arrogant scurrility and mendacity of those men seem to strike consonant chords in popular favor, for they reap a golden harvest, whilst the conscientious practitioner rarely attains to a financial mediocrity.

Turn your eyes as you will, and you will see a flaming advertisement of the wonderful virtues of some nostrum, and whether it were discovered by a native, a Hottentot, a Kickapoo Indian, or a clergyman, some so-called "doctor" is advertising it now, and some of these "doctors" have really had all the advantages of a good medical education, but have thrown off the restraints of the code, and become impostors. But instead of being frowned down by an enlightened public, they are received with open arms and encouraged with sympathy, patronage and money, as though they had escaped from the prison of a common enemy, which they teach their admiring friends "The Code of Ethics" really is, in a moral and intellectual point of view.

If these impostors on the credulity of their race, derived their supporters from the more ignorant classes, it would not be so surprising, but these only follow the example of those whom they consider wise; and men, otherwise respectable and intelligent, subscribe and allow their portraits to be affixed to certificates of remarkable, and even impossible cures, which were effected by this or that nostrum, after all the doctors had failed; and even clergymen, on whom we naturally look as examplars, not uncommonly, so far betray an ignorant want of that morality which deals only in known truth, as to subscribe to these certificates, and these gentlemen sometimes even vend such nostrums in their sacerdotal perigrinations, thus associating religicn with charlatanism, so that we have now, in fact, and will soon, perhaps, have in name, "Smith's Baptist Bitters," and "Jones' Methodist Elixir."

Neither national nor state legislators have ever shown any favors to the codists, as is exemplified in the Patent Office, where perjurers, who have never invented anything but falsehoods, can patent the ideas and prescriptions of better men, and by the insufficiency of the laws which the various legislatures have passed.

But on the contrary, some of these law givers have even trodden the code and its adherents, contemptuously and contemptibly under foot, by endowing Keeley institutes; and as these legislators live, politically, on personal popularity, they act in accordance with their views of popular sentiment. So it follows that the people, as a mass, are more friendly to the irregular practice of medicine than they are to regular practice, according to "The Code of Ethics."

The average newspaper,—that boasted educator of our race,—is almost sustained by the advertisements of irregulars, and is therefore engaged in educating the public mind in the interests of charlatanism. A leading paper of this State, for a long time, offered an irregular Domestic Practice of Medicine as a premium to its subscribers, which was, of course, an endorsement by the paper of such a work and of the depraved conditions which give existence to such a work. And entirely too many medical journals are devoted to leaders and contributions in which the subject matter is only the gossamer robe of an advertisement of the impossible achievements of some proprietary medicine.

It is without the scope of this paper to discuss the shortcomings and misdoings of regular doctors themselves, although some who profess to follow the code, follow it at too great a distance, for some bad men will get into the best institutions, and some will get bad after getting into them; but in this respect we have the questionable consolation that doctors are perhaps no worse than other professional men.

Volumes could be filled with the sickening details of the many nefarious tricks and schemes of hydra-headed charletanisms to degrade the medical doctor and defraud him of his just patronage and dues, and the same volumes could show that these tricks and schemes are only too often successful in their objects, because the people are easily and willingly deluded by them.

It is clear then that regular physicians can look to no one but themselves to remedy the evils which so thickly beset them, and to restore the profession to its pristine dignity and prestige, and it is almost equally clear that only unremitted and vigorous enforcement of the principles of the code of ethics would have prevented the semi-disrepute into which the medical profession has fallen, for unfortunately the real worth of the profession is estimated by the many, by the worthlessness of its enemies in the guise of doctors, as the people seem to be unable to discriminate between the true and the false.

The remedy then lies in the removal of the ethical code from the cold atmosphere of passive acquiescence in which it now lies dormant, into the more genial warmth of enthusiasm, where it would assume the aggressive, and where it would become more active in the defense of its friends and the destruction of its enemies.

Concert of action is indispensible to success in this matter. and traitors to the cause must be carefully and vigorously eliminated. But if every physician and every candidate for graduation in medicine were organized into a society in which the subscribing to the Hyppocratic oath before a notary public was made a prerequisite to membership, the profession would soon command and not solicit that respect which it so justly merits. For an expulsion—for cause—from such a society would be tantamount to a conviction of perjury before the law, and no man could materially harm the fraternity under such conditions.

Such views may be too visionary for a realistic age like this, but if some way is not found to enforce the principles embodied in the Hyppocratic oath in matters pertaining to medicine as an art and science, the more honest and worthy practitioners will remain shut up in the "Code of Ethics," like the defenders of a besieged castle whose walls may be impregnable, but cannot prevent starvation.

